# Interleukin-1 receptor-associated kinase-1 is a therapeutic target for gastric cancer

**DOI:** 10.1007/s12672-025-03658-x

**Published:** 2025-10-14

**Authors:** Hui Shi, Jie Zhang, Xiaohua Bao

**Affiliations:** Department of Gastroenterology, The 964th Hospital of People’s Liberation Army Joint Logistic Support Force, No. 4799 Xi’an Road, Changchun, P.R. China

**Keywords:** IRAK1, Gastric cancer, Pacritinib, Xenograft model

## Abstract

Interleukin-1 receptor-associated kinase 1 (IRAK1), a pivotal mediator in innate immunity and inflammatory processes, exhibits constitutive activation across various cancers. Our study highlights the significant role of IRAK1 in gastric cancer progression. We observed elevated IRAK1 expression in both gastric cancer tissues and cells, which correlates with malignant phenotypes, including heightened proliferation, colony formation, and migratory capacity in normal gastric cells upon IRAK1 overexpression. Silencing IRAK1 genetically in gastric cancer cells effectively suppressed these oncogenic traits. Notably, we found that gastric cancer cells display marked sensitivity to IRAK1/4 inhibitor and pacritinib, the latter targeting JAK2 and IRAK1 specifically, over the pan-JAK inhibitor tofacitinib. In vivo experiments using a xenograft mouse model demonstrated the efficacy of pacritinib in arresting gastric cancer growth without eliciting significant systemic toxicity. Immunohistochemical analyses further confirmed pacritinib’s ability to attenuate tumor progression by inhibiting IRAK1 activity. Collectively, our results underscore IRAK1 as a promising therapeutic target in gastric cancer. Furthermore, the pharmacological blockade of IRAK1 by pacritinib, an established drug for myelofibrosis and severe thrombocytopenia treatment, holds potential for repurposing in gastric cancer therapy.

**Supplementary Information:**

The online version contains supplementary material available at 10.1007/s12672-025-03658-x.

## Introduction

Gastric cancer, primarily adenocarcinomas, accounts for approximately 90% of cases and ranks as the fifth most common malignancy globally, constituting the fourth leading cause of cancer-related deaths [1]. The current standard treatment for advanced gastric adenocarcinoma involves a combination of perioperative chemotherapy and radical surgical resection [2]. Advancements in understanding the genetic basis of gastric cancer have spurred the development of targeted therapies. Trastuzumab, a monoclonal antibody targeting human epidermal growth factor receptor 2 (HER2), is used for gastric cancer patients exhibiting HER2 overexpression or amplification [3]. Additionally, ramucirumab, a recombinant IgG antibody targeting vascular endothelial growth factor receptor 2 (VEGFR2), has been approved for use either as monotherapy or in combination with paclitaxel in the treatment of advanced and metastatic gastric cancer [4]. However, despite these interventions, the overall survival rates remain low for patients with gastric cancer, particularly in advanced stages.

IL-1 receptor-associated kinase 1 (IRAK1) is a critical signaling molecule involved in various biological processes, particularly in the innate immune response. As a member of the IRAK family, IRAK1 plays a central role in the Toll-like receptor (TLR) and interleukin-1 receptor (IL-1R) signaling pathways, where it mediates downstream signaling cascades leading to the activation of NF-κB and MAPK pathways [5, 6]. Beyond its immune-related functions, IRAK1 has also been implicated in cancer progression and development. Aberrant expression and dysregulation of IRAK1 have been observed in various cancer types, including breast, lung, prostate, melanoma, nasopharyngeal cancers and leukemia [7–12]. The overexpression of IRAK1 in cancer is often associated with poor prognosis and patient outcomes [13, 14]. In cancer cells, elevated levels of IRAK1 contribute to tumor growth, survival, invasion, metastasis, and resistance to chemotherapy and immunotherapy [7, 8]. Additionally, IRAK1 has been implicated in the modulation of the tumor microenvironment, promoting an immunosuppressive and pro-tumorigenic milieu. Targeting IRAK1 signaling pathways presents a promising therapeutic strategy for cancer treatment, and ongoing research efforts aim to elucidate its precise role and potential as a therapeutic target in cancer therapy. Expression of IRAK1 pathway genes is altered in patients with gastric cancer.

In this study, we systematically examined the expression profile of IRAK1 in gastric cancer relative to normal tissues and elucidated its significance in the progression of this disease. Furthermore, we investigated the therapeutic potential of IRAK1 inhibition through both genetic and pharmacological approaches, both in vitro and in vivo, shedding light on its efficacy as a treatment strategy for gastric cancer.

## Materials and methods

### Human tissue specimens and immunohistochemistry

Tissue samples from patients newly diagnosed with gastric cancer were collected at Department of Tissue Repository of the 964th Hospital of People’s Liberation Army Joint Logistic Support Force. Each sample, along with its corresponding adjacent tissue, underwent pathological confirmation post-operation. Paraffin wax-embedded tissue samples were sectioned. Deparaffinization and hydration were achieved by successive incubations in xylene and decreasing concentrations of ethanol solution, the slides were washed with PBS. Subsequently, they were fixed in 4% paraformaldehyde for 15 min, air-dried, and treated with 0.5% Triton X-100 for 20 min. PBS served as the negative control throughout the procedure. The slides were then incubated with IRAK or p-IRAK1 (T209, Abcam) and thoroughly washed with PBS. Following this, biotinylated rabbit anti-mouse IgG was applied. The slides were then incubated with DAB at room temperature, followed by counterstaining with haematoxylin and eosin (H&E). Slides were examined under light microscopy. Staining quantification was performed using ImageJ software, and the results were analyzed in a blinded manner.

### Cells and compounds

Human gastric epithelial cell lines, including the GES-1 cell line and various gastric cancer cell lines, were procured from the Beijing Institute of Cancer Research. These cell lines were cultured in Dulbecco’s modified Eagle’s medium (DMEM) supplemented with 10% heat-inactivated fetal bovine serum (Thermo Fisher Scientific) and Penicillin-Streptomycin (Sigma) within a 5% CO2 atmosphere. Additionally, primary normal human gastric epithelial cells (HGAEPC) were sourced from Cell Applications Inc. and cultured using GI Epithelial Cell Defined Culture Medium as per the manufacturer’s recommendations. Pacritinib, IRAK1/4 inhibitor and tofacitinib (Selleckchem) were reconstituted dimethyl sulfoxide.

### SiRNA knockdown

Cells were seeded into 6-well plates at a density of 2 × 10⁵ cells per well and allowed to adhere overnight. For siRNA-mediated knockdown, cells were transfected with 100 nM of IRAK1-targeting siRNA (Sense 5’-GTCAGAGCCACCGCAGATTAT-3’; Antisense 5’-ATAATCTGCGTGGCTCTGAC-3’) or scrambled negative control siRNA (siControl, GenePharma) using Lipofectamine 3000 reagent (Invitrogen) according to the manufacturer’s protocol. After 6 h, the transfection medium was replaced with fresh complete medium, and cells were incubated for an additional 48 h. The efficiency of IRAK1 knockdown was confirmed by Western blot analysis.

### Transient overexpression

Cells were seeded into 6-well plates at a density of 2 × 10⁵ cells per well and cultured overnight to reach 70% confluency. Transient overexpression of IRAK1 was performed by transfecting 2.5 µg of plasmid DNA per well using Lipofectamine™ 3000 reagent (Invitrogen), according to the manufacturer’s instructions. The plasmid construct was generated by cloning the full-length human IRAK1 coding sequence (NCBI Reference Sequence: NM_001569.4) into the pCMV3-C-his expression vector (Sino Biological). Cells were harvested 48 h after transfection for downstream assays. The efficiency of IRAK1 overexpression was confirmed by Western blot analysis.

### ELISA

The level of IRAK1 was assessed utilizing the Human IRAK-1 ELISA Kit (Abcam) with either tissue or cell lysates. Proteins were standardized to a concentration of 10 mg/ml using PBS. Subsequently, 50 µl of protein lysates or serially diluted human IRAK1 standards were added to microplates coated with antibodies against IRAK1. Incubation buffer served as the zero standard. Absorbance at 450 nm was measured on Microplate Reader (Tecan Infinite).

### Cell proliferation assay

Cell proliferation was assessed using the BrdU Cell Proliferation Assay Kit (Abcam, ab126556) following the manufacturer’s protocol. Cells were seeded in 96-well plates at a density of 5,000 cells per well in 100 µl complete medium. Following transfection or drug treatment, cells were incubated for 72 h before BrdU labeling. Absorbance was measured at 450 nm using a microplate reader (Tecan Infinite M200), and background was subtracted.

### Cell viability assay

Cell viability was determined using the CellTiter-Glo^®^ Luminescent Cell Viability Assay (Promega, G7571). Cells were seeded in 96-well plates at 5,000 cells per well and treated with pacritinib. After 24 h, 100 µl of CellTiter-Glo reagent was added directly to each well, and luminescence was recorded using a Tecan Infinite M200 plate reader. Results were normalized to vehicle-treated controls. Experiments were repeated three times in triplicate.

### Cell migration assay

Cell migration was evaluated using the CytoSelect™ 24-Well Cell Migration Assay (Cell Biolabs, CBA-100) according to the manufacturer’s instructions. A total of 1 × 10⁵ cells in 300 µl of serum-reduced medium (2% FBS) were seeded into the upper chamber, while the lower chamber contained 500 µl of medium with 10% FBS as a chemoattractant. After an 8-hour incubation at 37 °C, cells that migrated through the membrane were stained, lysed, and quantified by absorbance at 560 nm. Three fields per well were imaged using an Olympus IX71 microscope and quantified using ImageJ (version 1.53t).

### Colony formation assay

Anchorage-independent colony formation was assessed using a soft agar assay. The bottom layer consisted of 1.5 ml of 0.7% Bacto agar (BD Difco) in complete medium poured into 6-well plates and allowed to solidify. Cells (5000 per well) were suspended in 1.5 ml of 0.3% agar mixed with complete medium and plated as the top layer. Cells were incubated at 37 °C in 5% CO₂ for 2 weeks. Fresh medium (100 µl) was added twice per week. Colonies were imaged under a light microscope (Olympus CKX53), and colonies larger than 50 μm were counted manually using ImageJ software. The average number of colonies was determined from three independent experiments.

### Real time PCR

Cellular RNA extraction was conducted utilizing the Fastagen200 kit, followed by reverse transcription into cDNA using a Double-Strand cDNA Synthesis kit. Reverse transcription was performed using a Double-Strand cDNA Synthesis Kit (Takara) to generate cDNA from 1 µg of total RNA. Quantitative real-time PCR using SYBR Green PCR Master Mix (Takara) on an ABI StepOnePlus Real-Time PCR System (Applied Biosystems). The cycling conditions were: 95 °C for 5 min, followed by 40 cycles of 95 °C for 15 s and 60 °C for 30 s. Relative gene expression was calculated using the 2^−ΔΔCt method, with GAPDH as the internal control. Primer sequences for IRAK1 and GAPDH were presented in Table S1.

### Animal experiments

The experimental procedures involving mice were approved by the Institutional Animal Care & Use Committee. In brief, a cell suspension containing 10^7 cells was prepared, washed, resuspended in 0.1 ml of complete DMEM, and then subcutaneously injected into the right flanks of 4-week-old male BALB/C nude mice. The animals were monitored daily for tumor development. Following one week, mice bearing tumors were randomly assigned to receive either vehicle (0.5% methylcellulose and 0.1% Tween-80 in H2O) or oral pacritinib treatment at a dose of 40 mg/kg. Tumor tissues were subsequently excised, fixed in 10% formalin, and processed for standard immunohistochemistry.

### Statistical analyses

All experiments were performed in at least three independent biological replicates, unless otherwise stated. Data are presented as mean ± standard deviation. Statistical comparisons between groups were performed using two-tailed unpaired Student’s t-test or one-way ANOVA followed by Tukey’s post hoc test. A p-value of < 0.05 was considered statistically significant. Statistical analyses were conducted using GraphPad Prism 9.

## Results

### IRAK1 upregulation is a feature of gastric cancer and confers malignant cell properties on normal gastric cells

**Fig. 1:**
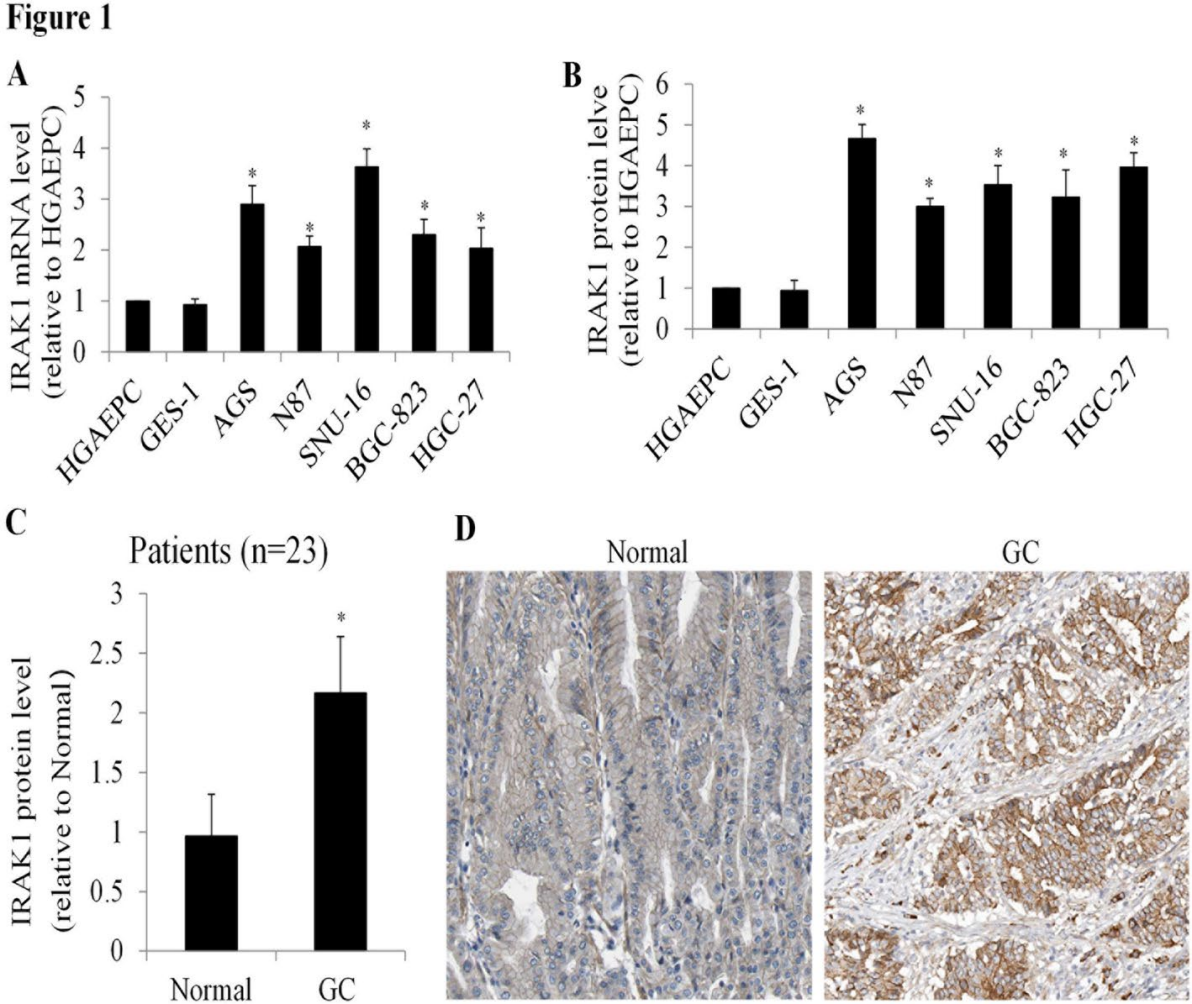
IRAK1 Is Activated in Gastric Cancer and IRAK1 Overexpression Enhances Normal Gastric Cell Functionality. (**A**) mRNA and (**B**) protein levels of IRAK1 in normal gastric cells (HGAEPC and GES-1) and gastric cancer cells (AGS, N87, SNU-16, BGC-823, and HGC-27). IRAK1 expression is significantly elevated in gastric cancer cells compared to normal gastric cells. ELISA assay was applied for quantification of IRAK1 protein across various types of cells. (**C**) ELISA quantification of IRAK1 levels in gastric cancer and adjacent normal gastric tissues (*n* = 23). (**D**) Representative immunohistochemistry of IRAK1 in malignant and adjacent normal tissues from a patient with newly diagnosed gastric cancer. Scale bar represents 50 μm. For mRNA level analysis, IRAK1 level was normalized with β-actin. For ELISA analysis, IRAK1 level was normalized with total protein amount. *, *P* < 0.05, compared to normal control

We firstly performed transcriptional expression analysis on cell total RNA obtained from a diverse panel of gastric cancer cell lines, capturing the heterogeneity in gene expression patterns. This panel also included two normal gastric cell types: one immortalized normal cell line and primary normal cells. Our analysis revealed a consistent 2- to 3.6-fold increase in IRAK1 mRNA levels across all tested gastric cancer cell lines compared to normal gastric cells (Fig. 1A). Consistent with transcriptional analysis, by using ELISA analysis on cell lysates, we observed in the same gastric cancer cell lines that the significant increase in IRAK1 protein level (Fig. 1B). Expanding our investigation, we performed ELISA analysis of IRAK1 on paired samples of gastric cancer tissue and adjacent normal gastric tissue obtained from 23 patients newly diagnosed with gastric cancer. The clinicopathological characteristics of these patients, including age, TNM stage, tumor sites, and histology, were summarized in Table 1. Our analysis revealed a consistently higher IRAK1 protein level in gastric cancer tissues compared to normal gastric tissues (Fig. 1C). This upregulation of IRAK1 was further confirmed through immunohistochemistry examination of patient samples, where elevated IRAK1 levels were observed in gastric cancer tissues (Fig. 1D). These comprehensive analyses collectively indicate that upregulation of IRAK1 is a common feature of gastric cancer.

**Table 1:** Clinicopathological features of gastric cancer patients

Patient Number	Age	Tumor sites	Histology	TNM stage
GC#1	54	Cardia	Unknown	II
GC#2	36	Cardia	Intestinal	II
GC#3	64	Non-cardia	Diffuse	III
GC#4	60	Non-cardia	Diffuse	I
GC#5	58	other	Intestinal	I
GC#6	49	other	Diffuse	II
GC#7	49	Cardia	Diffuse	II
GC#8	78	Non-cardia	Diffuse	III
GC#9	81	Non-cardia	Mixed	I
GC#10	75	other	Diffuse	II
GC#11	54	Non-cardia	Diffuse	II
GC#12	36	Cardia	Intestinal	I
GC#13	64	Cardia	Intestinal	III
GC#14	76	Non-cardia	Mixed	I
GC#15	56	Non-cardia	Unknown	II
GC#16	61	other	Intestinal	II
GC#17	72	Non-cardia	Diffuse	III
GC#18	53	Cardia	Intestinal	II
GC#19	76	Non-cardia	Unknown	II
GC#20	51	Cardia	Diffuse	III
GC#21	53	Non-cardia	Intestinal	III
GC#22	59	Cardia	Unknown	I
GC#23	69	Non-cardia	Intestinal	II

Tumour, node and metastasis (TNM) stage; other include Site overlapping and unspecified. Mixed is of both intestinal and diffuse

**Fig. 2:**
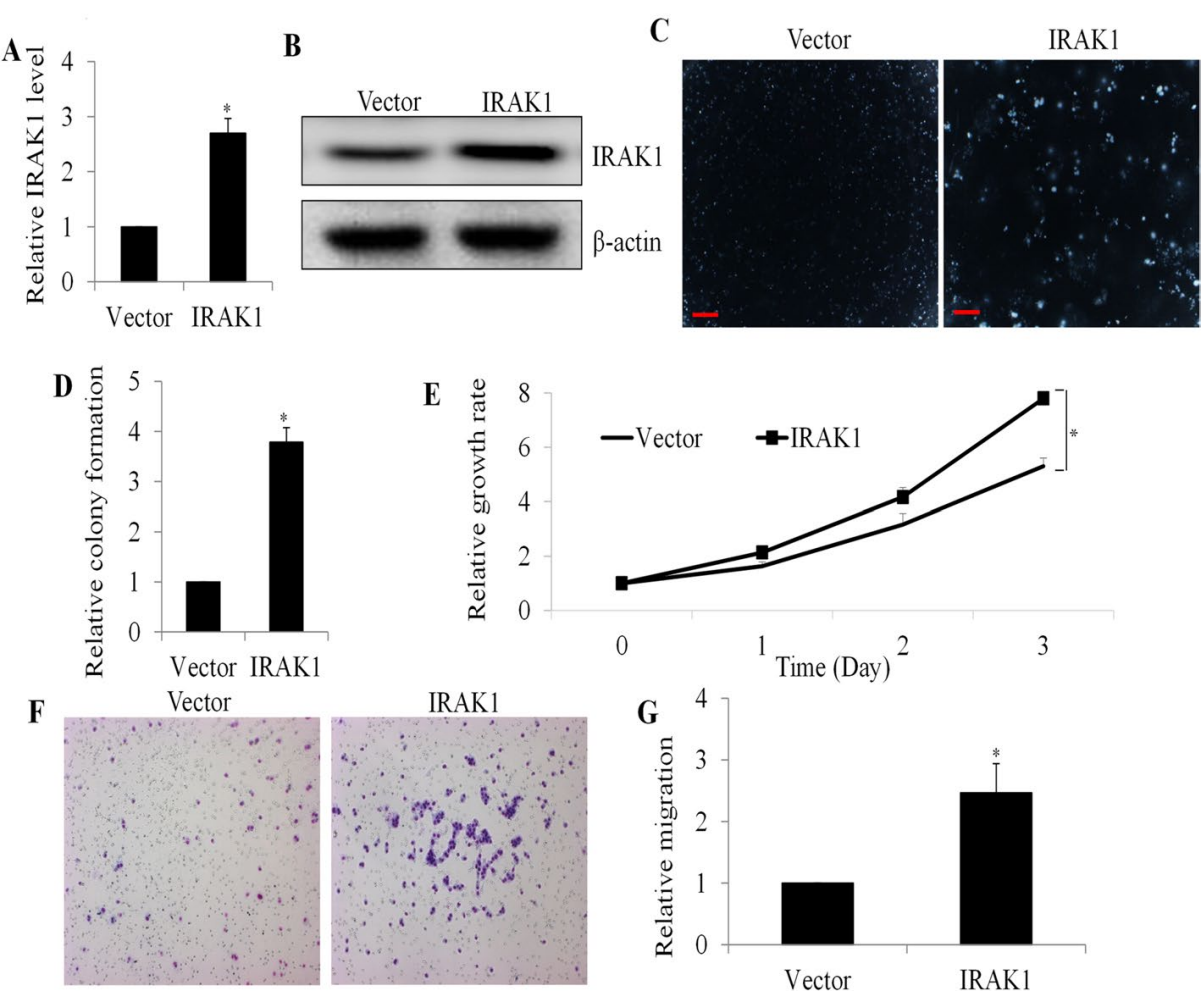
IRAK1 Overexpression Enhances Normal Gastric Cell Functionality. (**A** and **B**) Significant elevation of IRAK1 protein levels in GES-1 cells following IRAK1 overexpression. (**C** and **D**) Significant increase in colony formation in GES-1 cells with IRAK1 overexpression. Representative images of colony formation are presented. Scale bar represents 25 μm. (**E**) Enhanced proliferation and (**F** and **G**) migration capabilities in GES-1 cells upon IRAK1 overexpression. Colony and migrated cell counts were performed under a microscope. *, *p* < 0.05, compared to Vector control

Malignant cells are usually exhibited with increased cell mobility, invasive growth and cell cycle acceleration. We used colony formation, migration and proliferation assays to determine if IRAK1 overexpression is able to confer malignant cell properties on normal gastric cells. We transfected GES-1 cells with a cytomegalovirus-based vector expressing IRAK1, resulting in approximately a 2.5-fold increase in IRAK1 expression compared to the vector control (Fig. 2A and B). Subsequently, we observed a significant increase in colony formation, with IRAK1-overexpressing GES-1 cells forming approximately four times as many colonies compared to controls (Fig. 2C and D). Consistent with expectations, we found that IRAK1 overexpression led to a 1.5-fold increase in the growth rate of GES-1 cells (Fig. 2E) and a 2.5-fold increase in the number of migrated cells (Fig. 2F and G). These demonstrate that upregulation of IRAK1 overexpression confers malignant properties on normal gastric cells.

### Targeting IRAK1 inhibits gastric cancer cell activities

**Fig. 3:**
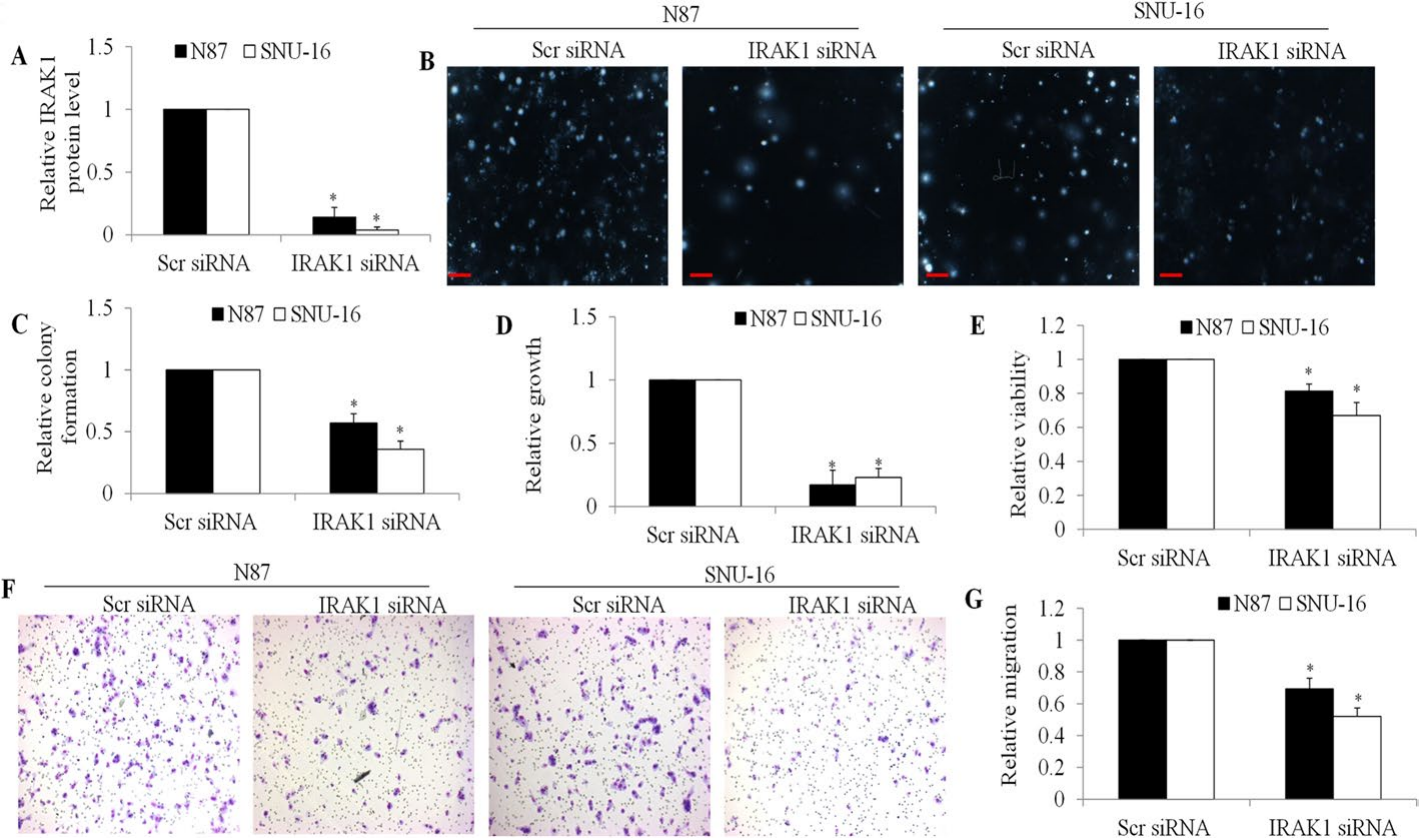
Suppression of Gastric Cancer Activities by IRAK1 siRNA Knockdown. (**A**) Reduced IRAK1 protein levels observed in cells transfected with IRAK1 siRNA. IRAK1 knockdown significantly attenuated colony formation (**B** and **C**), hindered growth (**D**), decreased viability (**E**), and reduced migration (**F** and **G**) in N87 and SNU-16 cells. All assessments were conducted 24 h post-transfection. Scale bar represents 25 μm. *p < 0.05, compared to siRNA control

We explored the therapeutic potential of targeting IRAK1 in gastric cancer using a loss-of-function approach in N87 and SNU-16, which exhibit the lowest and highest expression levels of IRAK1 among the tested lines. These two models were therefore chosen to represent the range of IRAK1 expression levels and to assess the functional impact of IRAK1 knockdown across different expression contexts. Gastric cancer cells were transfected with specific siRNA, leading to an approximately 90% reduction in IRAK1 protein expression (Fig. 3A and Figure S1). Our investigation revealed that IRAK1 depletion resulted in a significant decrease in colony formation, proliferation, viability, and migration in gastric cancer cells (Fig. 3B to G). Notably, among these cellular activities, IRAK1 depletion had the most pronounced impact on gastric cancer cell growth, while exhibiting a lesser impact on cell viability.

### Pacritinib inhibits gastric cancer cell activities

**Fig. 4:**
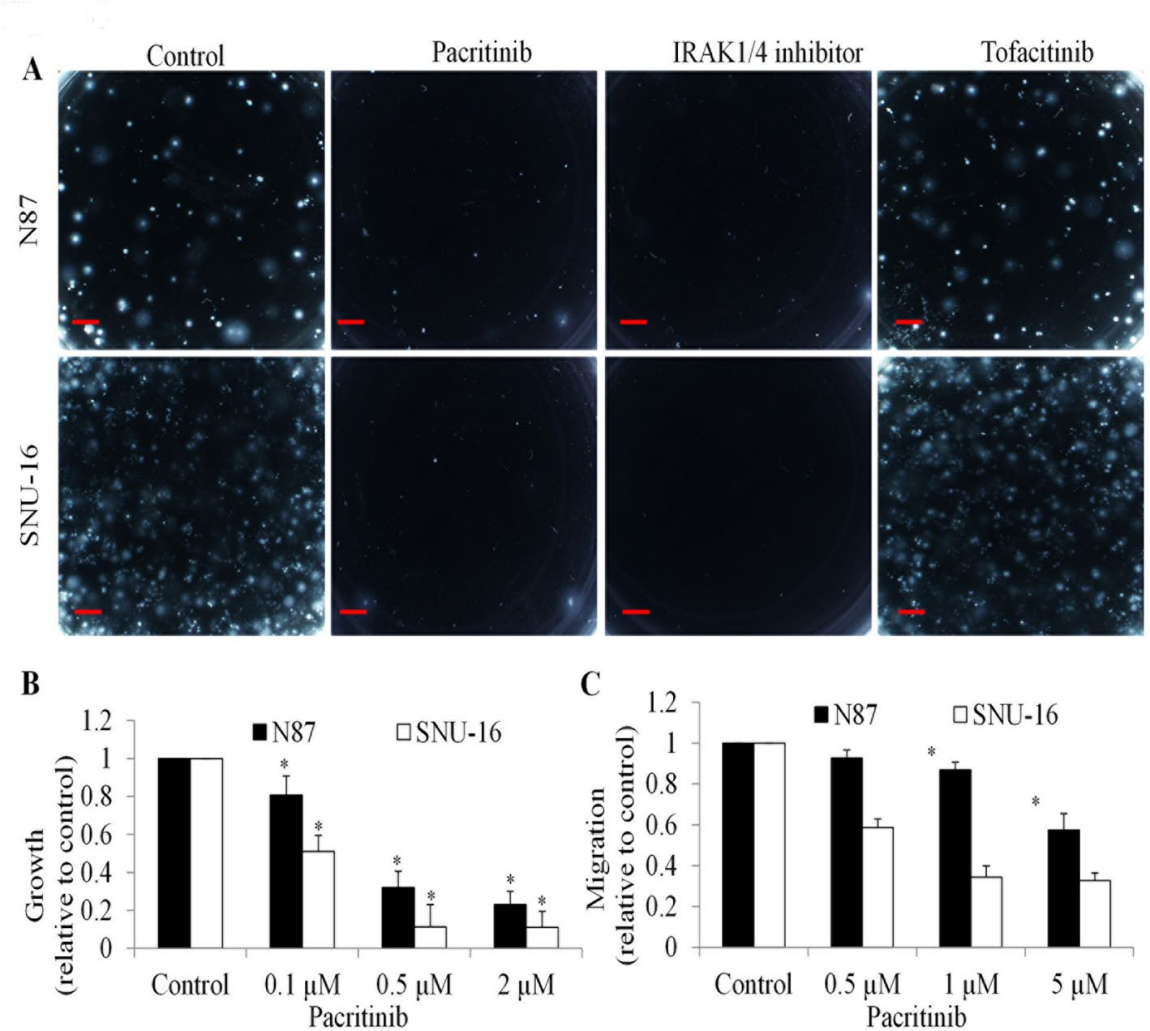
Pacritinib Inhibits Gastric Cancer Cell by Suppressing IRAK1 Activity In Vitro. (**A**) Representative images of colony formation in N87 and SNU-16 cells treated with 1 µM pacritinib, IRAK1/4 inhibitor or tofacitinib. Scale bar represents 25 μm. Pacritinib significantly decreased proliferation (**B**) and migration (**C**) in gastric cancer cells. *, *p* < 0.05, compared to control

Pacritinib has been identified as a kinase inhibitor of targeting both JAK2 and IRAK1 [7, 15]. To assess whether pacritinib can be used to treat gastric cancer through inhibiting IRAK1, we investigated the effects of pacritinib, IRAK1/4 inhibitor and tofacitinib on gastric cancer cells. IRAK1/4 inhibitor is an inhibitor of IRAK1- and IRAK4 and tofacitinib is a pan-JAK inhibitor. Our findings demonstrate that pacritinib effectively reduced colony formation in both N87 and SNU-16 cells (Fig. 4A). Similarly, the IRAK1/4 inhibitor exhibited notable suppression of colony formation, whereas tofacitinib did not yield such effects. These results suggest that pacritinib’s efficacy in inhibiting gastric cancer cells may primarily involve IRAK1 rather than JAK2. Furthermore, our study revealed that pacritinib dose-dependently decreased proliferation and migration in gastric cancer cells (Fig. 4B and C). Consistent with IRAK1 knockdown, pacritinib treatment resulted in a moderate reduction in cell viability (Figure S2).

### Pacritinib inhibits gastric cancer growth and IRAK1 activity in mice

**Fig. 5:**
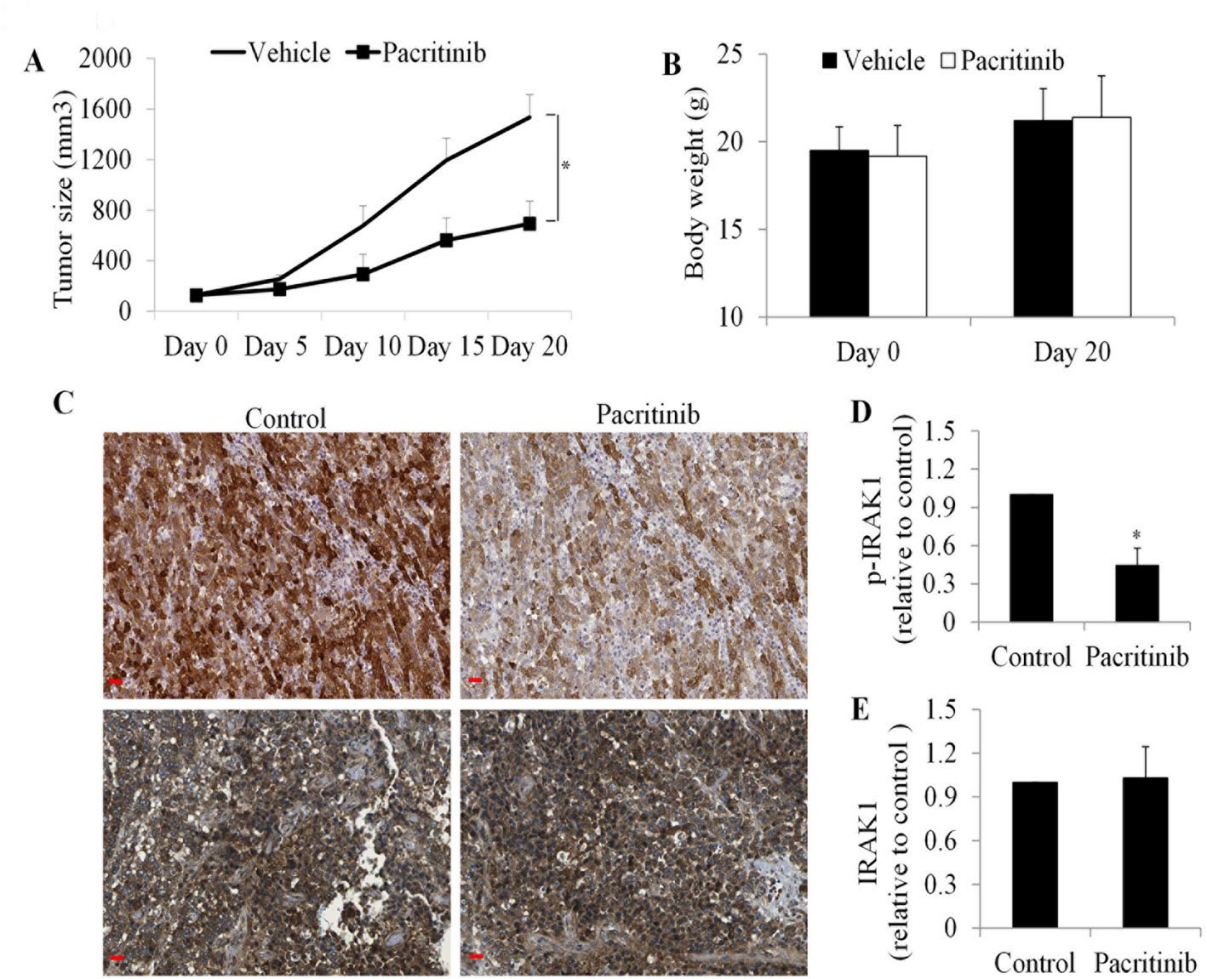
Pacritinib Inhibits Gastric Cancer Growth and Suppresses IRAK1 Activity In Vivo. (**A**) Significant reduction in N87 tumor growth observed in mice treated with pacritinib. (**B**) Comparable body weights recorded between control and pacritinib-treated groups of mice. (**C** to **E**) Levels of phospho-IRAK1 and total IRAK1 in subcutaneous tumors treated with vehicle control or pacritinib. Representative immunohistochemistry images are displayed. Scale bar represents 25 μm. Quantification of phosphor-IRAK1 and total IRAK1 staining was performed using Image J. *, *p* < 0.05, compared to control

To evaluate the effect and underlying mechanism of pacritinib in gastric cancer in vivo, we utilized SNU-16 cells to establish xenograft tumors in nude mice. Within a week of cell implantation, palpable tumors had developed, at which point mice were divided into groups receiving either vehicle or pacritinib treatment. Our findings revealed that pacritinib effectively reduced tumor growth, with a noticeable decrease observed as early as 5 days after treatment initiation. This inhibitory effect was sustained throughout the duration of treatment (Fig. 5A), ultimately resulting in a 60% reduction in tumor growth by day 20. Importantly, pacritinib administration did not induce any significant changes in mice body weight, indicating its non-toxic nature (Fig. 5B). Immunohistochemical analysis revealed a marked reduction in phosphorylated IRAK1 (p-IRAK1) levels in tumors from pacritinib-treated mice (Fig. 5C and D). Notably, total IRAK1 expression remained largely unchanged (Fig. 5C and E), indicating that pacritinib selectively inhibited IRAK1 activation without altering overall protein expression.

## Discussion

We present here IRAK1 as a therapeutic target for gastric cancer based on comprehensive expression analysis of patient tissues and functional assessments in preclinical disease models. Notably, we identify pacritinib as a potential treatment agent for gastric cancer. Pacritinib is an attractive candidate as it is already available for clinical use in the treatment of myelofibrosis and thrombocytopenia [16]. It has also been shown to be active against various cancer types, including leukemia, colon and rectal cancer, glioblastoma and lung cancer [17–20]. Moreover, ongoing clinical trials are investigating its effectiveness in chronic myelomonocytic leukemia, prostate cancer and relapsed T-cell lymphoproliferative neoplasms and (NCT04858256, NCT06218628, NCT04635059 and NCT06159491). Our research lays the groundwork for initiating clinical trials to evaluate pacritinib’s efficacy in patients with gastric cancer.

Our research initially uncovered the overexpression of IRAK1 in gastric cancer tissues and cells compared to their normal counterparts, indicating that IRAK1 upregulation is a common characteristic of gastric cancer. This finding aligns with Reza et al.’s observation that the expression of IRAK1 pathway genes (e.g., IRAK4) is altered in the serum of gastric cancer patients with Helicobacter pylori infection [21]. Notably, elevated IRAK1 expression has been consistently associated with poor prognosis. For example, patients with hepatocellular carcinoma exhibiting high IRAK1 expression have a shorter overall survival time compared to those with low IRAK1 expression [22]. Similarly, glioma patients with overexpressed IRAK1 have shown unfavorable prognoses [23]. Moreover, elevated IRAK1 expression correlates positively with reduced overall survival, distant metastasis-free survival, and relapse-free survival rates [8]. Our functional analysis further supports these findings, indicating that IRAK1 overexpression confers malignant properties onto normal gastric cells. This is consistent with previous research showing IRAK1’s role in promoting aggressive growth, metastasis, and acquired resistance in triple-negative breast cancer [8], as well as its association with paclitaxel resistance in nasopharyngeal carcinoma [7]. Given this body of evidence, we hypothesize that IRAK1 overexpression could serve as a valuable prognostic marker for gastric cancer patients.

Consistently, our experiments demonstrated that inhibiting IRAK1 in gastric cancer cells using siRNA led to reductions in growth, migration, and colony formation, indicating the potential therapeutic benefit of targeting IRAK1 in gastric cancer. Moreover, we showcased the efficacy of pacritinib, an inhibitor of both JAK2 and IRAK1, in pre-clinical models of gastric cancer. Importantly, we established that pacritinib acts specifically on gastric cancer cells by inhibiting IRAK1 rather than JAK2. This was supported by the finding that inhibition of IRAK1/4, but not the JAK inhibitor tofacitinib, elicited similar effects to pacritinib. Furthermore, our in vivo studies revealed that pacritinib reduced IRAK1 activity, as evidenced by the decreased levels of phosphorylated IRAK1 observed in tumors from mice treated with pacritinib. This underscores the potential of pacritinib to target IRAK1-driven mechanisms of resistance in cancer, as documented in prior research [6–8]. Given these promising results, it is prudent to investigate the efficacy of pacritinib in models of resistant gastric cancer.

In summary, our findings highlight IRAK1 overexpression as a prevalent characteristic of gastric cancer, underscoring the potential of targeting IRAK1 as a promising therapeutic avenue. Our results demonstrate the efficacy of IRAK1 inhibition through pacritinib in both in vitro and in vivo models of gastric cancer.

## Supplementary Information

Supplementary Material 1

## Data Availability

This study did not generate or analyze datasets that require deposition in a public repository. All data supporting the findings of this study are included within the published article and its supplementary information files.
