# Supplementary material for: Interleukin-1 receptor-associated kinase-1 is a therapeutic target for gastric cancer
Source: Discov Oncol. 2025 Oct 14;16:1882. doi: 10.1007/s12672-025-03658-x (PMC12521082; doi:10.1007/s12672-025-03658-x)
Supplement: Supplementary file 1 — Supplementary Material 1 [file 12672_2025_3658_MOESM1_ESM.docx]

**Interleukin-1 receptor-associated kinase-1 is a therapeutic target for gastric cancer**

Figure S1. Western blot analysis of total IRAK1 in gastric cancer cells after siRNA depletion.

Figure S2: Pacritinib treatment decreases gastric cancer cell viability. *, p < 0.05, compared to control.

| Gene | Forward Primer (5’→3’) | Reverse Primer (5’→3’) |
| --- | --- | --- |
| IRAK1 | TCAGCTTTGGGGTGGTAGTG | TAGATCTGCATGGCGATGGG |
| GAPDH | GGAGCGAGATCCCTCCAAAAT | GGCTGTTGTCATACTTCTCATGG |

Table S1: Primer sequences used for quantitative real-time PCR. Forward and reverse primers for IRAK1 and the housekeeping gene GAPDH are listed in the 5’ to 3’ orientation.

Original images of Western blots for Figure 2B.

Original images of Western blots for Figure S1.
